# Formation and elimination of soluble fibrin and D-dimer in the bloodstream

**DOI:** 10.3325/cmj.2023.64.421

**Published:** 2023-12

**Authors:** Anastasiia Udovenko, Yevgen Makogonenko, Daria Korolova, Nadiya Druzhyna, Volodymyr Chernyshenko, Serhiy Komisarenko

**Affiliations:** 1 Department of Protein Structure and Function, Palladin Institute of Biochemistry of NAS of Ukraine, Kyiv, Ukraine; 2 Department of Molecular Biology, Palladin Institute of Biochemistry of NAS of Ukraine, Kyiv, Ukraine

## Abstract

Soluble fibrin is composed mainly of desA fibrin and fibrinogen oligomers consisting of fewer than 16 monomers partially cross-linked by factor XIIIa. Soluble fibrin cannot stimulate Glu-plasminogen activation by tissue plasminogen activator (t-PA); therefore, it may not be a direct predecessor of D-dimer. However, within the microcirculatory system, soluble fibrin oligomers may form microclots. Fibrin microclots stimulate Glu-plasminogen activation by t-PA, a process resulting in the formation of Glu-plasmin. Glu-plasmin dissolves the microclots, forming D-dimer. In normal and pathological blood plasma samples, soluble fibrin levels are substantially higher than those of D-dimer. Their concentrations in the plasma are also regulated by transendothelial transfer, absorption by blood macrophages, and binding and internalization with low-density lipoprotein receptors of the cells of the reticuloendothelial system. Therefore, the exact mechanisms of fibrin clots formation and elimination in normal and pathological conditions remain unclear. In this study, we reviewed findings on the molecular mechanisms of the formation and dissolution of fibrin clots, fibrin-dependent activation of Glu-plasminogen by t-PA, and blood plasma behavior in the microcirculatory system. Finally, we proposed a model that explains the relations of D-dimer and soluble fibrin underlying the common and separate mechanisms of their formation and elimination.

The hemostasis system supports the integrity of the blood circulatory system necessary for blood to perform its homeostatic function (1). If a vessel is disrupted, the hemostasis system is locally and briefly activated; it seals the “hole” and the damaged tissue with a thrombus and then returns to a state of rest (2-4).

The thrombus performs a protective function and later serves as the matrix for regenerating the damaged vessel and tissues. The surface of the thrombus facing the blood flow is blocked by fibrinogen (Fg) molecules. This is why the thrombus cannot be accessed by the fibrinolytic system, which would otherwise activate and prematurely destroy the thrombus by plasmin (5,6).

Hemostasis is misregulated in various conditions, such as infectious, cardiovascular, and oncological diseases. Its involvement in the pathological process complicates the course of the disease and requires special attention (7). Thus, unless hemostasis is activated to terminate bleeding and restore tissue integrity, it can be viewed as a component of the body's overall response to some pathological process (4,8). In the former case, the thrombus possesses an important physiological function; in the latter, the system becomes an “instrument” of the pathology and often complicates the disease course.

The blood plasma coagulation system is activated during the inflammatory process accompanying any pathology; the primary vehicle is tissue factor (TF) in microvesicles, which initiates the extrinsic blood coagulation pathway (7).

Soluble fibrin (SF) is both the main molecular marker of activation of the blood coagulation system and its product (9,10). SF exists in the form of fibrin-Fg complexes and short oligomers, in which desA fibrin molecules are partially covalently cross-linked by factor XIIIa and are blocked at the sticky ends by Fg molecules (9). Fibrin desA oligomers cannot stimulate plasminogen activation by tissue plasminogen activator (t-PA) (11). However, under the microcirculation conditions, the blood flow slows down by two to three orders of magnitude, so fibrin oligomers can bind to the glycocalyx and form microclots (fibrils). Thus, in this environment, D-dimers are mainly produced from SF. On the surface of microclots, a ternary complex of Glu-plasminogen (Glu-Pg), fibrin, and t-PA is formed. In this complex, Glu-Pg is activated by t-PA to form Glu-plasmin, which dissolves microclots (microthrombi), a process resulting in the formation of D-dimer (10,12). Accordingly, D-dimer is considered a marker of activation of coagulation and fibrinolytic processes.

The presence of fibrin and D-dimer in the blood plasma and their ratio are important predictive markers of thrombotic complications in various diseases and surgical interventions (13-16). However, despite the close functional connection, there is no direct correlation between the SF and D-dimer concentrations in the blood plasma. The aim of this study was to examine in more detail the molecular mechanisms of formation and elimination of SF and D-dimer to find out which of them were involved in the regulation of the concentration of these important molecular markers of the state of the hemostasis system.

## General characteristics of the hemostasis system

The hemostasis system includes plasma and cellular components of blood (endothelial cells of vessel walls, platelets, red blood cells, and other blood cells) (1-3,17,18). The basis of the hemostasis system are the coagulation system and the fibrinolytic system.

**Figure 1:**
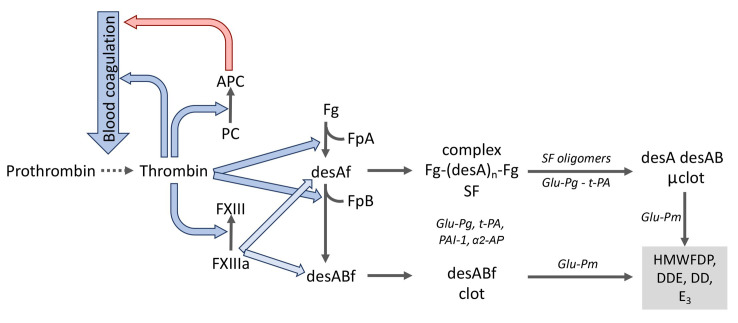
Molecular interactions in the activation of blood coagulation, and related fibrinolytic and protein C systems. Fg – fibrinogen; FXIII, FXIIIа – fibrin-stabilizing factor XIII and its activated form; PС – protein C, APC – activated protein C; FpA, FpB – fibrinopeptides A and B; desAf, desABf – fibrin desA and fibrin desAB; complex Fg-(desA_n_)-Fg – fibrinogen with fibrin desA oligomers complex; SF – soluble fibrin; desA desAB μclot – fibrin desA and desAB clot cross-linked by FXIIIa as microclots in capillaries; α2-AP – α2-antiplasmin; PAI-1 – type I plasminogen activator inhibitor; Glu-Pg – Glu-plasminogen; Glu-Pm – Glu-plasmin; HMWFDP – high-molecular weight fibrin degradation products; DD – D-dimer; DDE – complex of D-dimer and E_1_-fragment; Е_3_ – the product of degradation by plasmin of fragment E with cleaved BβN-domains.

The coagulation system includes a number of plasma proteins, such as coagulation factors and inhibitors, and platelets. The fibrinolytic system, which includes Glu-Pg, t-PA proenzymes, α2-antiplasmin (α2-AP), and plasminogen activator inhibitor-1 (PAI-1) is functionally related to the coagulation system. It removes fibrin deposits from vessel walls and microclots from the blood plasma. Other compounds of hemostasis are the kallikrein-kinin system, protein C anticoagulation system, and thrombin activatable fibrinolysis inhibitor (TAFI) (9,12,17). A unified model of the functioning of the hemostasis system includes the participation of cellular and protein factors of blood coagulation in the formation and dissolution of blood clots (Figure 1) (2,18,19).

**Figure 2:**
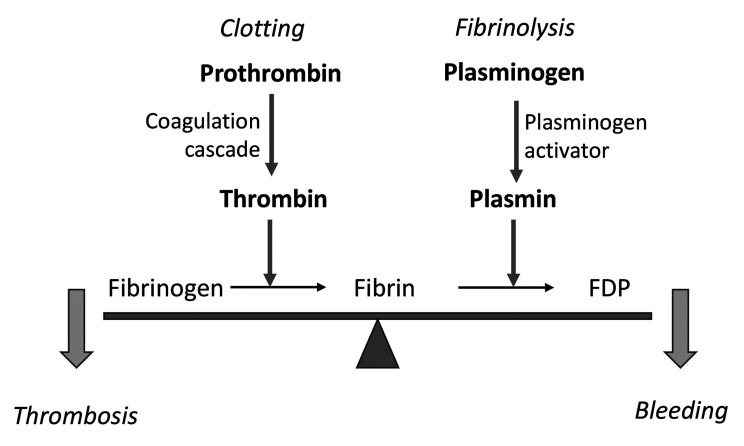
The balance between blood clotting and fibrinolysis. Activation of blood clotting system leads to thrombin formation, while plasmin is being formed on the surface of the clot to dissolve it and fibrin degradation products (FDP) are formed. A disruption of this balance leads to the intravascular clotting or bleedings (10).

 It is generally accepted that the existence of functionally opposite processes of coagulation and fibrinolysis in the hemostasis system indicates a balance between the formation and destruction of fibrin clots in the blood plasma (Figure 2) (9,10). However, medical practice has shown that most pathologies associated with thrombus formation and bleeding can be cured by correcting disorders in the clotting system without the regulation of fibrinolysis (20). Also, the endogenous fibrinolytic system is not very effective in the case of heart attacks, thromboembolisms, and strokes because the mechanisms of activation of coagulation and fibrinolysis are not simultaneous but sequential. Therefore, in these diseases, administering even ultrahigh concentrations of fibrinolytic agent (t-PA) with a delay of three hours is ineffective (21-23).

Normally, when vessel walls are damaged and tissues are destroyed, the hemostasis system is activated by the plasma FVIIa and TF complex, originating from damaged vessel walls and the surrounding tissues (7,8). In various diseases, even when there is no damage to blood vessels and body tissues, the FVIIa + TF complex activates the blood coagulation system. This complex is contained in procoagulant microparticles in the blood plasma stimulated by inflammatory factors accompanying any pathology (7,8,24,25). Microvesicles containing TF are formed and released into the blood by platelets, endothelial cells, monocytes, and other blood cells. TF is also found in the products of cell necrosis. In addition, the proenzymes of the intrinsic pathway of the coagulation system can be activated by kallikrein, metalloproteinases, and other blood proteases (4,19,26-29). The activation of the hemostasis system results in the formation of thrombin – the central enzyme of the hemostasis system. Thrombin accelerates the activation of the coagulation system due to the activation of FV, FVIII, and FXI according to the positive feedback mechanism. It is also responsible for the formation of polymeric fibrin – the basis of the thrombus. Furthermore, it activates protein C, TAFI, and, indirectly, the fibrinolytic system (30-32). In the case of damage, the hemostasis system is activated with the participation of the extrinsic pathway of the coagulation system. In the case of diseases, the activation process includes the extrinsic and, to a lesser extent, intrinsic coagulation pathways (4).

## Fibrinogen and formation of soluble fibrin

The forms of fibrin produced during damage to vessels and body tissues and those produced during various diseases are significantly different from each other. In the former, due to the high rate of local activation of thrombin in damaged areas of vessels, platelets are being activated, and Fg is quickly transformed into the desAB fibrin mesh. The mesh locally strengthens the thrombus that closes the damaged area (9,10). In diseases, especially at the initial stage, thrombin is activated systemically and slowly. Thrombin is formed in the blood plasma in sub- and nanomolar concentrations, which leads to the transformation of Fg into desA fibrin in very small concentrations and results in the formation of SF. The self-assembly of desA fibrin into protofibrils and further into a clot is inhibited in the plasma by a high concentration of Fg. As a result, complexes of short fibrin desA protofibrils with Fg (known as SF) are formed (33-35). The presence of SF in the plasma, simultaneously with other pathological factors, is believed to create the danger of intravascular thrombus formation, which can lead to strokes, thromboembolism, and heart attacks. Therefore, the presence of SF in the plasma is an important diagnostic indicator of the state of the hemostasis system, and its level indicates the degree of involvement of the hemostasis system in the pathological process (13-16).

Fibrin desA oligomers that compose SF consist of fewer than 16 monomers each. The oligomers are formed through the interaction of A:a and C:c polymerization sites (36). Each strand of the oligomer is partially cross-linked by FXIIIa along the C-ends of the γ-chains, which are in end-to-end contact with the neighboring D regions of fibrin molecules. The “sticky” ends of oligomers bind Fg molecules due to the interaction of A:a and, possibly, C:c polymerization sites. This terminates further elongation and leads to the formation of protofibrils, their lateral association, and the formation of fibrils (microclots). Fibrin desA oligomers bind components of the fibrinolytic system – Glu-Pg and t-PA; however, they are unable to stimulate the activation of Glu-Pg with the formation of Glu-plasmim (11), which breaks down the clot to end-products: D-dimer, D, and E3 fragments (37). The presence of γ'-chains (427 amino acid residues long, weakly crosslinked by FXIIIa) in ~8% of the D regions of fibrin molecules prevents the formation of γ-γ stitches in ~16% of the neighboring fibrin molecules, which affects the final concentration of D-dimer (38).

## Activation of Glu-Pg by t-PA, degradation of fibrin clot, and formation of D-dimer

**Figure 3:**
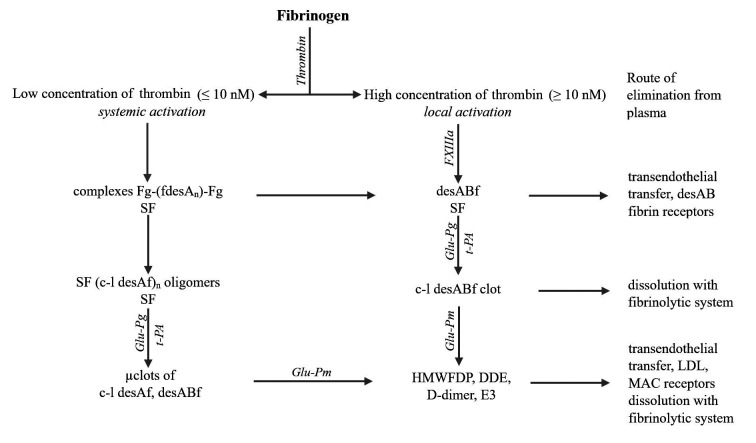
The ways of formation, dissolution, and removal of soluble desA, desAB fibrins, their clots, and fragments of polymeric fibrin, including D-dimer. desAf, desABf – fibrin desA and fibrin desAB; SF – soluble fibrin; Fg – fibrinogen; FXIIIа – activated form of fibrin-stabilizing factor XIII; SF (c-l desAf)_n_ oligomers – soluble fibrin cross-linked desA oligomers; complexes Fg-(fdesA_n_)-Fg – fibrinogen with fibrin desA oligomers complexes; c-l desABf clot – fibrin desAB clot cross-linked by FXIIIa circulating in blood plasma; desAf μclot – fibrin desA clot cross-linked by FXIIIa as microclots in capillaries; HMWFDP – high-molecular weight fibrin degradation products; DDE – complex of D-dimer and E_1_-fragment; Е3 – the product of degradation by plasmin of fragment E with cleaved BβN-domains; Glu-Pg – Glu-plasminogen; Glu-Pm – Glu-plasmin; t-PA – tissue-type plasminogen activator.

SF concentration indicates the activation level of the coagulation system in the blood and depends on the concentration of thrombin constantly being formed in the plasma (Figure 3) (13). It is generally accepted that the coagulation and fibrinolysis systems of the blood plasma are in a dynamic equilibrium (10), which suggests a connection between the concentration of SF and D-dimer as indicators of the activation of both systems. However, literature data and our previous findings do not support this assumption (39,40). For example, a previous study (35) found a correlation between SF and D-dimer determined by ELISA methods. However, the methods used did not allow distinguishing between the newly formed SF and D-dimer as a product of cross-linked clots breakdown. Another important issue is the big difference in the rates of SF (3 μg/mL under normal conditions and 10-100 μg/mL under pathological conditions) and D-dimer (0.05 μg/mL under normal conditions and 1-5 μg/mL under pathological conditions). The discrepancy in the concentrations of SF and D-dimer and the lack of correlation between the concentrations of SF and D-dimer in the blood plasma indicate different underlying mechanisms of regulation. Let us discuss the possible ways in which D-dimer can originate from SF accumulation. Theoretically, D-dimer can arise directly from the SF destroyed by the fibrinolytic system. However, studies that investigated the composition and structure of SF and biochemical mechanisms of fibrin clot formation and destruction indicate that partially cross-linked fibrin desA oligomers in the complex with Fg are not able to stimulate the activation of Glu-Pg, which is bound to them, by t-PA (11). The structures that stimulate Glu-Pg activation are formed at the next stage of fibrin polymerization – the stage of lateral association of protofibrils, where the fibrillar structure of the clot is formed (11,30). At the same time, Glu-Pg and t-PA molecules are localized on the surface of fibrils, where they form the triple activator complex Glu-Pg-fibrin-t-PA, and the degradation of the clot begins on the surface of fibrils. Therefore, SF in the oligomeric form cannot directly serve as a source of D-dimer but must first turn into a fibrillar form of fibrin – a microclot.

**Figure 4:**
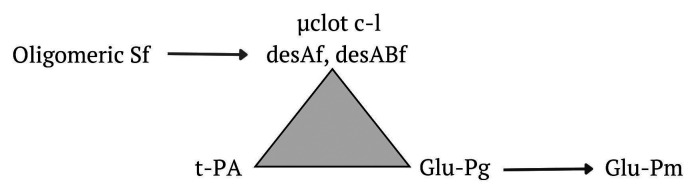
Formation and degradation of a fibrin clot noncrosslinked and crosslinked by FXIIIa polymeric fibrin. Oligomeric SF – oligomeric soluble fibrin; μclot c-l – microclot cross-linked by FXIIIa in capillaries; desAf, desABf – fibrin desA and fibrin desAB; t-PA– tissue-type plasminogen activator; Glu-Pg – Glu-plasminogen; Glu-Pm – Glu-plasmin.

Fibrin microclots are not formed in large vessels, where the speed of blood flow and the size of the vessels ensure intensive mixing of the blood. They are most likely formed in the vessels of the microcirculation system, where the flow rate decreases to a few millimeters per second, and the ratio of the surface area of the endothelium to the blood volume increases by several orders of magnitude. The layer of endothelial cells lining the bed of all blood vessels is ~ 10^13^ cells. Its weight is 1.5 kg, and its surface area reaches from 4000 to 7000 m^2^ (41). As a result, there is an increased probability that SF oligomers will come into contact with the surface of the capillary walls and with each other, a process resulting in the formation of fibrillar structures of polymeric fibrin – microclots. On the surface of fibrin desA in the BβN-domain, there is a heparin-binding site that can facilitate SF's interaction with the endothelium glycocalyx (42). It was found that 40% of the endothelial surface in the vessels of the microcirculation system belonged to arterioles and venules. Also, more than 90% of t-PA activity appears in arterioles and venules, and only 3% – in capillaries (41,43). However, it can be assumed that the contacts of thrombin-containing SF with the capillary wall contribute to the transfer of thrombin to thrombomodulin on the surface of endothelial cells and stimulation of the synthesis and release of t-PA into the capillary lumen. A simple calculation shows that, in the lumen of the capillary, the concentration of t-PA can reach hundreds of units of activity per milliliter of blood. These factors can contribute to the formation of a ternary activator complex on a microclot that was formed from SF. The activation of Glu-Pg into Glu-plasmin is followed by the destruction of the microclot to the final product – DDE. They are, after cleavage of βN domains (β 1 (15)-42 β-chain peptides) in the circulatory system, further destroyed to D-dimers and E_3_ fragments (42,44) (Figure 4).

According to the concept of a balance in the hemostasis system, the amount of polymeric fibrin formed by the activated coagulation system and the amount of polymeric fibrin removed by the fibrinolysis system should be interrelated. The initial steps of degradation of polymeric desAB fibrin in a clot that is uncrosslinked by FXIIIa and a clot crosslinked by FXIIIa are regulated by identical mechanisms (37,45).

In a fibrin clot crosslinked by FXIIIa, Glu-plasmin, after splitting off αC-regions, cleaves α-, β- and γ-chains of the fibrin molecule in the hinge regions with the formation of large fragments of polymeric fibrin and the final product – DDE. This fact indicates that B:b polymerization sites interact within the same protofibrils, as suggested by Doolittle (46). Therefore, K42-A43 peptide bonds in the Bβ-chain of polymeric cross-linked fibrin are difficult for plasmin to reach. DDE is the precursor of D-dimer (9,37). Further cleavage of 1 (15)-42 polypeptide fragments of B(β)N-domains of E regions in DDE leads to the disintegration of the complex into the final fragments – D-dimers and E_3_ fragments. These data indicate that a direct relationship between the concentration of SF and the concentration of D-dimer is impossible. A mandatory intermediate link in this chain is the fibrin microclot.

The structure of SF oligomers differs considerably from the structure of protofibrils of polymeric fibrin. This difference is due to the huge excess of Fg over desA fibrin concentration; the absence of B polymerization sites in the desA fibrin molecule; and the presence of αC-region in a complex with the BβN-domain in the desA fibrin molecule (33,44). However, the mechanisms of degradation of the structure of normal fibrils or protofibrils and SF oligomers *in vitro* are similar: first, Glu-plasmin cleaves the αC regions and then 1-42 fragments of the BβN domains of the E regions of molecules. After that, it sequentially cleaves α-, β-, and γ-chains of the molecule in the hinge region, which leads to the formation of terminal products: D-dimer, D, and E_3_ fragments of polymeric fibrin. Since FXIIIa is not able to completely cross-link D regions in SF, due to the presence of γ'-chains in ~8% of Fg molecules, the concentration of the formed D-dimer will potentially be lower than the concentration of SF. This also contributes to the lower concentration of D-dimer compared with the initial SF in the blood plasma.

 The concentrations of E_3_ fragment and D-dimer must correlate with each other. In this case, the E_3_ fragment can be a marker of the state of the hemostasis system, just like the D-dimer. The ratio of D-dimer and E_3_ fragments indicates the intensity of formation and destruction of fibrin clots crosslinked and uncrosslinked by FXIIIa in the blood plasma and can be a marker of the stability of any fibrin clot.

 It is interesting to consider the preventive and predictive aspects of the diagnostic parameters SF and D-dimer tests from the 4 P (predictive, preventive, personalized, participatory) medicine point of view. We consider the SF parameter to be more important, since it indicates early manifestations of the blood factors that activate prothrombin conversion into thrombin (TF-containing microparticles, kallikrein, neutrophil extracellular traps, activated platelets, atherosclerotic changes in the endothelial wall of blood vessels) and SF formation. A stable elevated SF level indicates the presence of a pathological locus in the patient's body and the need for a more in-depth examination.

 The D-dimer parameter indicates the disease progression, which leads to the formation of fibrin clots in the blood plasma and on the blood vessels walls. Although these early thrombi are available for destruction by the endogenous fibrinolytic system, there is a need for early prophylactic anticoagulation therapy.

Important and independent factors regulating the concentration of SF and D-dimer are their transfer through the endothelial layer of the vessel walls into the intercellular space; absorption by blood cells; and internalization with the participation of MAC-1 and LDL- receptors of reticuloendothelial system cells (47-53). The half-life of desA and desAB fibrin, Fg, and D-dimer in the bloodstream is 2, 8, 8, and 9 (15) hours, respectively. However, the specific inputs of these components of the blood flow environment into removing fibrinogen, SF, D-dimer, and Fg hydrolysis products (X, Y, D, and E fragments) have yet to be elucidated (54) and require further consideration.

## Conclusions

 SF, as a marker of activation of the coagulation system, and D-dimer, as evidence of the presence of cross-linked fibrin deposits in the cardiovascular system and a marker of their destruction by the fibrinolytic system, are functionally interconnected. Their connecting link is fibrin clot. However, the mechanisms of fibrin formation during tissue damage and those during diseases are significantly different. In the case of tissue damage, fibrin is formed locally and leads to the formation of a thrombus, whereas in the case of diseases, the conversion of Fg to fibrin occurs systemically. Systemic fibrin formation maintains a higher-than-normal level of SF in the blood plasma, which, together with other pathological factors, creates the danger of intravascular clotting.

 This is why different forms of fibrin are formed during injuries and diseases: fibrin desAB, which forms insoluble clots in places of tissue damage, and fibrin desA, which forms SF in the plasma. SF consists of oligomers of desA fibrin that can be crosslinked by FXIIIa along the C-terminal sections of the γ-chains. The “sticky” ends of oligomers bind Fg molecules, forming SF monomeric complexes. Fibrin desA oligomers bind Glu-Pg and t-PA but cannot stimulate the activation of Glu-Pg with the resulting formation of Glu-plasmin, which dissolves the clot. Therefore, SF in the oligomeric form cannot serve as a source of D-dimer but must first turn into a fibrillar form of fibrin – a microclot.

 Despite the dynamic equilibrium between the coagulation and fibrinolytic systems in the blood plasma, there is no correlation between the concentration of SF and D-dimer as indicators of the activation of the coagulation and fibrinolytic systems of plasma. The profound difference in SF and D-dimer concentrations and the lack of correlation between SF and D-dimer accumulation in the blood plasma indicate different regulation mechanisms of the formation and elimination of these markers of the state of the hemostasis system.
